# The epidemiology of post-traumatic stress disorder in Norway: trauma characteristics and pre-existing psychiatric disorders

**DOI:** 10.1007/s00127-016-1295-3

**Published:** 2016-10-18

**Authors:** Eva Lassemo, Inger Sandanger, Jan F. Nygård, Knut W. Sørgaard

**Affiliations:** 1 SINTEF Technology and Society, Health Research, Trondheim, Norway; 2 University of Tromsø, Tromsø, Norway; 3 Cancer Registry of Norway, Oslo, Norway; 4 Nordland Hospital, Bodø, Norway

**Keywords:** Post-traumatic stress disorder, Trauma, Epidemiology, Gender differences, Pre-existing psychiatric disorders

## Abstract

**Purpose:**

The prevalence of PTSD differs by gender. Pre-existing psychiatric disorders and different traumas experienced by men and women may explain this. The aims of this study were to assess (1) incidence and prevalence of exposure to traumatic events and PTSD, (2) the effect of pre-existing psychiatric disorders prior to trauma on the risk for PTSD, and (3) the effect the characteristics of trauma have on the risk for PTSD. All stratified by gender.

**Method:**

CIDI was used to obtain diagnoses at the interview stage and retrospectively for the general population *N* = 1634.

**Results:**

The incidence for trauma was 466 and 641 per 100,000 PYs for women and men, respectively. The incidence of PTSD was 88 and 31 per 100,000 PYs. Twelve month and lifetime prevalence of PTSD was 1.7 and 4.3 %, respectively, for women, and 1.0 and 1.4 %, respectively, for men. Pre-existing psychiatric disorders were risk factors for PTSD, but only in women. Premeditated traumas were more harmful.

**Conclusion:**

Gender differences were observed regarding traumatic exposure and in the nature of traumas experienced and incidences of PTSD. Men experienced more traumas and less PTSD. Pre-existing psychiatric disorders were found to be risk factors for subsequent PTSD in women. However, while trauma happens to most, it only rarely leads to PTSD, and the most harmful traumas were premeditated ones. Primary prevention of PTSD is thus feasible, although secondary preventive efforts should be gender-specific.

## Introduction

Disabling psychopathology attributable to distressing experiences is very old and has been referred to in the literature throughout time, albeit under different names. Descriptions of combat fatigue, which fit diagnostic criteria of the present day PTSD remarkably well, can be found in Greek and Norse literature, as well as in Shakespeare [1–3]. Exposure to potentially traumatic events (PTEs) is common [4, 5], yet only a fraction of people exposed ever meet the current diagnostic criteria for PTSD [6]. Men are consistently more frequently exposed to traumatic events than women [5, 7–11], but studies consistently report higher lifetime prevalence rates of PTSD in women [5, 12]. PTEs are events that may be perceived and experienced by the individual as life-threatening or dangerous. Such events are component causes for the PTSD diagnosis—i.e., necessary, but not sufficient.

Disaster psychiatry and traumatic stress research have traditionally been focused on specific PTEs, natural or human caused, rather than on the occurrence of trauma in the general population [6]. Therefore, the knowledge of occurrence of traumas and subsequent risk of PTSD in the general population is scarce. In particular, knowledge is lacking on how the experience of different traumas and the subsequent risk of PTSD varies by gender.

Therefore, we aimed to assess: (1) incidence and prevalence, lifetime and 12 month, of exposure to PTEs and PTSD in a sample representative of the general population, (2) the effect of pre-existing depressive-, anxiety-, and/or somatoform disorders prior to potentially traumatic exposure on the risk for subsequent PTSD, and (3) the effect the characteristics of potential traumas have on the subsequent risk for PTSD. All stratified by gender.

## Method

### Study design and setting

This study is a cross-sectional population sample with retrospective data collection. The data stemmed from the longitudinal population OsLof study examining mental health in an urban (Oslo) and a rural (Lofoten) community. Statistics Norway (SSB) drew a random, representative sample of 5000 individuals ages 18 and above in 1989 (*T*_0_) and a random supplement sample of 1000 in 2000 (*T*_1_). Of 2727 potentially eligible individuals at *T*_0_, 2014 (74 %) were interviewed and thus became study participants. Of these, 1300 (64 %) participated at *T*_1_. From the 2000 (*T*_1_) supplement, 525 individuals were eligible, resulting in 391 (74 %) additional study participants. Hence, the cohort at *T*_1_ consisted of a total of 1691 individuals, of which 1300 were recruited at *T*_0_ and 391 recruited at *T*_1._ In this study, retrospective, dated information about past and present mental health problems, obtained by the CIDI at *T*_1_, were used.

The following exclusion criteria for this study were used: missing CIDI interview, not having completed interview, or missing sex, resulting in 1634 subjects interviewed at *T*_1_ and used for the present research. The OsLof study and its study population are presented in detail in Sandanger et al. [13].

### Measures

To obtain accurate diagnoses based on ICD-10 criteria, an updated electronic version—CIDI-M 1.1, of the Composite International Diagnostic Interview (CIDI) [14] was used. The CIDI is a circumstantial procedure in that every symptom is not only asked, but the answers are probed by severity and dated: “Did you tell a doctor about (actual symptom)? Did you tell another professional about (actual symptom)? Did you take medication more than once for (actual symptom)? and Did (actual symptom) interfere with your life and activities a lot?” Once one of these questions is answered with “yes,” the rest are omitted and the symptom is considered important enough to be investigated further. The CIDI then rules out symptoms likely caused by drugs, medication, alcohol or somatic illness alone. Only psychiatric symptoms are kept. Age at onset for the first and last episodes of disorder/symptom was recorded along with date of event. All disorders with full positive criteria were included. The CIDI maps symptoms throughout the respondents’ lifetime up to the date of interview.

#### Post-traumatic stress disorder (PTSD)

Post-traumatic stress disorder (ICD-10 code F43.1) was included. Exposure to a traumatic event was recorded through the CIDI interview. The worst episode of traumatic exposure was recorded along with time of exposure. Caseness of fulfilling diagnostic criteria was reported as PTSD.

A traumatic event is “an experience that causes physical, emotional, psychological distress or harm” [15]. It is an event that is perceived and experienced as life-threatening, or as a significant threat to physical or psychological well-being by the individual. As identical events may have severely different impact from person to person, the term ‘potentially traumatic event’ is used to convey this heterogeneity.

#### Depression

Affective and depressive disorders (ICD-10 codes F31.4–F34.1) and organic depressive disorder (ICD-10 code F06.32) with onset prior to PTSD diagnosis were included.

#### Anxiety

Anxiety disorders (ICD-10 codes F40.0–F41.9) with onset prior to PTSD diagnosis were included.

#### Somatoform disorders

Somatoform disorders (ICD-10 codes F45.0–F45.9) with onset prior to PTSD diagnosis were included.

#### Categorization of trauma

PTEs were categorized into accidental (war event, natural catastrophe, serious accident, witnessing PTEs happen to others, and verbal threat/violence from non-close relation) and premeditated (physical threat (weapon), rape, sexual abuse as a child, imprisoned, taken hostage or kidnapped, and verbal threat/violence from close relation).

### Statistical analysis

Data were analyzed using survey data commands svy for STATA. Proportions of women and men exposed to trauma and PTSD, and having pre-existing psychiatric disorders, were tested for independence using Pearson’s Chi-square. All tests were done at the 95 % confidence level. All results were weighted by gender and age to reflect the composition of the Norwegian population in 2000, i.e., adjusting them for difference in response rate. Age was categorized into four groups; 18–34, 35–49, 50–65, and 66+ .

Incidence was reported as the number of new cases per 100,000 person years (PYs). Two measures of prevalence were reported: first, as the share of the population with prevalent PTSD in their lifetime (up to interview) and second, as the share of the population with prevalent PTSD in the 12 months preceding the interview.

Associations between pre-existing psychiatric disorders and trauma and PTSD were analyzed by an odds ratios (OR) with 95 % confidence intervals (95 % CIs) and Kaplan–Meier survival estimates. Categories of trauma and PTSD were analyzed by an odds ratios (OR) with 95 % confidence intervals (95 % CIs).

#### Imputation

Approximately one-third of those having experienced trauma (none of the PTSD cases) had failed to report the age at which their worst trauma occurred. Hence, cold-deck class mean imputation [16], as a refinement of the overall mean imputation procedure, was employed to impute age at worst potentially traumatic exposure. Cases were categorized into classes based on gender, place, and trauma type. Little difference was unveiled between the raw mean age at worst trauma and that emerging from the imputation. However, between the classes based on gender, place, and trauma type, significant differences remained.

Statistical analyses were performed using STATA version 14.1.

## Results

### Incidence and prevalence of exposure to PTEs and PTSD by gender

In the sample, more men (*n* = 203, 26.2 %) than women (*n* = 186, 21.7 %) were exposed to PTEs (*p* = 0.031). Of those exposed to trauma, more women (*n* = 38, 20.4 %) than men (*n* = 10, 4.9 %) filled diagnostic criteria for PTSD (*p* < 0.001), yielding a lifetime prevalence of 4.4 and 1.3 %, and a 12-month prevalence of 1.8 and 0.8 % for women and men, respectively. Given that PTEs are component causes for the PTSD diagnosis—necessary, but not sufficient, no individuals will have PTSD without having experienced a trauma. The sample had an underrepresentation of persons 18–34 years of age and an overrepresentation of persons 35–65 years of age, as compared with the Norwegian population (data not shown).

Results showed that 25.9 % men and 20.6 % women were exposed to PTEs (*p* = 0.018). Of those exposed to trauma, more women (20.6 %) than men (5.6 %) filled diagnostic criteria for PTSD (*p* = 0.001), yielding a lifetime prevalence of 4.3 and 1.4 %, and a 12-month prevalence of 1.7 and 1.0 %, for women and men, respectively. The incidence of potentially traumatic exposures was 466 (95 % CI 398–549) and 641 (95 % CI 556–742) per 100,000 PYs for women and men, respectively. The incidence rate of PTSD was 88 (95 % CI 63–129) and 31 (95 % CI 13–67) per 100,000 PYs for women and men, respectively. The incidence rate ratio (IRR), for women to men, was 2.92 (95 % CI 1.39–6.17). There were no statistically significant differences between women and men being exposed to PTEs at an earlier age, filling diagnostic criteria for PTSD more rapidly, or having longer lasting episodes of PTSD (Table 1).

**Table 1:** Study population stratified on gender, trauma exposure, and pre-existing psychiatric diagnoses. Weighted by age and gender

	At risk for PTE (*N* = 1634)		At risk for PTSD (*N* = 389)	
	Women (*n* = 859)	Men (*n* = 775)	Women (*n* = 186)	Men (*n* = 203)
Proportion exposed to PTE/with PTSD (%)	20.6	25.9*	20.6*	5.6
Mean age at worst traumatic exposure (95 % CI)	24.1 (21.9–26.3)	23.0 (21.9–24.0)	20.2 (15.5–24.8)	19.3 (13.1–25.6)
Mean age at onset PTSD (95 % CI)			24.8 (20.1–29.6)	19.9 (13.3–26.5)
Mean years from worst traumatic exposure to PTSD (95 % CI)	–	–	4.7 (1.2–8.1)	0.5 (−0.3 to 1.3)
Mean years of duration of PTSD (95 % CI)	–	–	9.1 (5.3–12.9)	17.2 (6.9–27.6)
Psychiatric disorder pre-existing to trauma and PTSD				
None (%)	61.1	84.4*	36.4	92.9*
Depression	11.3*	4.2	19.7	0.0
Anxiety	16.9*	7.4	43.9*	7.1
Somatoform disorder	25.3*	9.5	44.9*	0.0
Any pre-existing disorder	38.9*	15.2	63.6*	7.1

Gender difference tested statistically

Results are weighted to the Norwegian population

* *p* < 0.05

Applying the 2015 Norwegian population age structures for 18 and older, an incidence rate for PTSD of 88 and 31 per 100,000 PYs for women and men, respectively, indicated an annual national incidence of approximately 1776 and 627 cases, respectively. An approximate 9405 women and 12,961 men were annually exposed to potentially traumatic events. During the year 2015, there would have been 34,309 and 20,220 prevalent cases of PTSD among women and men.

Despite a limited number of cases in this study, an indication of a pattern emerged when investigating incidence rates by 10-year age steps. Among men, a marked peak in the incidence rate of trauma appeared in the age group 20–30, and tapered off by age 60. However, no new cases of PTSD occurred beyond age 33. This pattern was less pronounced among women, where there was no incident PTSD beyond age 60.

### Pre-existing depressive-, anxiety-, and/or somatoform disorders prior to potentially traumatic exposure and the risk for subsequent PTSD

**Fig. 1:**
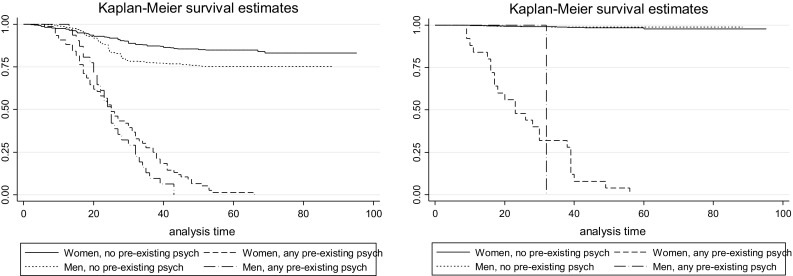
Kaplan–Meier survival curves depicting probability of experiencing potentially traumatic events (*panel 1*) and fulfilling PTSD diagnostic criteria (*panel 2*) if suffering from any pre-existing psychiatric disorder and not, for women and men. Time at risk from birth. Note: Results are weighted to the Norwegian population

The odds ratio (ORs) for conditional PTSD when suffering from pre-existing psychiatric disorders are presented, by gender, in Table 2. For women, any pre-existing depressive, anxiety or somatoform disorder was associated with an increased risk for PTSD [OR 3.6 (95 % CI 2.6–5.0)]. The absence of pre-existing psychiatric conditions gave a lower risk estimate for women [OR 0.3 (95 % CI 0.2–0.4)]. As shown in the Kaplan–Meier estimate (panel 1 of Fig. 1), men were exposed to PTEs earlier in life than women. The same was true for both those suffering from, and those not suffering from, any pre-existing psychiatric disorder. Panel 2 of Fig. 1 shows the steady rate at which women suffering from any pre-existing psychiatric disorder, compared to those not, developed PTSD. For women, pre-existing psychiatric disorder was associated with subsequent PTSD (*p* < 0.001). There were only a few men with pre-existing psychiatric disorders and traumatic exposure, and only one filled the diagnostic criteria for PTSD.

**Table 2:** Odds ratio (ORs) for post-traumatic stress disorder (PTSD) following exposure to traumatic event and pre-existing psychiatric disorders and by trauma type

	Subsequent PTSD			
	Women		Men	
	OR	95 % CI	OR	95 % CI
Pre-existing psychiatric disorder^a^				
Depressive disorder	2.5	(1.6–3.8)	–	
Anxiety disorder	7.0	(4.9–10.1)	0.9	(0.5–1.6)
Somatoform disorder	3.2	(2.3–4.4)	–	
Any disorder	3.6	(2.6–5.0)	0.3	(0.2–0.6)
No disorder	0.3	(0.2–0.4)	3.1	(1.8–5.1)
Trauma type				
Accidental	1	(ref)	1	(ref)
Premediated	57.0	(7.3–447.9)	5.6	(1.3–24.8)
Both	95.6	(11.0–831.3)	1.3	(0.2–7.6)

Weighted by age and gender

Results are weighted to the Norwegian population

^a^Disorder with an age at onset earlier than age at worst traumatic exposure

### Characteristics of potential traumas and the subsequent risk for PTSD

Women and men were exposed to PTEs of differing nature (Table 3). Women were, to a greater extent, exposed to rape (*p* < 0.001) and sexual abuse (*p* = 0.011). Within the “other” category, women were exposed to more verbal threats/violence from close relations (*p* = 0.017). Contrary, men were, to a greater extent, exposed to serious accidents (*p* < 0.001), imprisonment, and so on (*p* = 0.004), and witnessed traumatic events happen to others more frequently (*p* < 0.001). Within the “other” category, men were exposed to more verbal threats/violence from non-close relations (*p* = 0.042).

**Table 3:** Lifetime prevalence of exposure to potentially traumatic events (PTEs) and conditional post-traumatic stress disorder (PTSD) by gender

Traumatic event	At risk for PTE (*N* = 1634)		At risk for PTSD (*N* = 389)		P^a^ lifetime trauma exposure	P^1^ PTSD
	Women (*n* = 859) %	Men (*n* = 775) %	Women (*n* = 186) %	Men (*n* = 203) %		
Any traumatic event	20.6	25.9	20.6	5.6	0.018*♂	0.001*♀
1. War event	2.3	3.9	4.4	6.3	0.086	0.773
2. Physical threat (weapon)	6.4	8.1	33.5	2.8	0.218	<0.001*♀
3. Rape	3.2	0.1	54.5	0	<0.001*♀	–
4. Sexual abuse as a child	3.2	1.2	53.6	36.6	0.011*♀	0.450
5. Natural catastrophe	2.0	1.4	3.7	9.1	0.357	0.521
6. Serious accident	4.4	9.8	10.9	4.4	<0.001*♂	0.233
7. Imprisoned, taken hostage, kidnapped	0.8	3.1	24.6	7.3	0.004*♂	0.360
8. Witness above events happen to others	3.9	9.4	17.0	3.5	<0.001*♂	0.014*♀
9. Other	4.0	3.7	31.2	11.8	0.710	0.102
Verbal threat/violence from close relation	*2.6*	*0.9*	*40.4*	*38.0*	0.017*♀	*0.927*
Verbal threat/violence from non-close relation	*1.3*	*2.7*	*8.2*	*3.9*	0.042*♂	*0.598*
Two or more events	6.8	8.5	34.8	5.3	0.221	<0.001*♀
Accidental	41.8	56.1	1.7	30.3	0.010*♂	0.001*♂
Premediated	43.4	20.3	68.3	54.1	<0.001*♀	0.449
Both	13.8	22.3	30.0	15.7	0.043*♂	0.370

Trauma type given exposed to trauma and given PTSD caseness by gender. Weighted by age and gender

Results are weighted to the Norwegian population

^a^Fisher’s exact test

* Significant on the 95 % level

♀ Significantly different in direction of women

♂ Significantly different in direction of men

Although men more frequently were exposed to traumatic events, women were four times more likely to meet the criteria for PTSD. Of those exposed to potentially traumatic events, 20.6 % of women and 5.6 % of men met PTSD criteria (*p* = 0.001). The probability of meeting PTSD criteria was greater for women when exposed to a physical threat (*p* < 0.001) or witnessing traumatic events happen to others (*p* = 0.014). Men did not have significantly higher probability of PTSD for any particular traumatic exposure. Among women, rape, sexual abuse, and verbal threats/violence from close relations, and among men, sexual abuse and other events led to highest conditional risk for PTSD.

Women having experienced a premeditated potentially traumatic event had a significant increased probability of subsequent PTSD, as compared with women having experienced accidental PTEs [OR 57.0, 95 % CI (7.3–447.9)] (Table 2). Women having experienced both accidental and premediated traumas had further increased OR for PTSD, as compared with those having only experienced accidental traumatic events. For men, there was an increased probability [OR 5.6, 95 % CI (1.3–24.8)] of subsequent PTSD for those having experienced a premeditated potentially traumatic event, as compared with those having only experienced accidental traumatic events.

Both men and women had an increased probability of PTSD if exposed to two or more traumatic events, women more so (*p* < 0.001). However, no linear relationship between the number of potentially traumatic exposures and PTSD emerged (data not shown).

## Discussion

PTSD and traumatic exposure add considerably to the national burden of disease. With a retrospectively estimated incidence rate of 88 and 31 PTSD cases per 100,000 PYs for women and men, respectively, this is higher than that of, for example, colorectal cancer (25 and 28) or lung cancer (28 and 32) [17]. PTSD differs from the major somatic non-communicable diseases (NCDs) (cardiovascular disease, most cancers, diabetes, and chronic respiratory disease) in that it largely appears during the young adult age range. In cases where PTSD is not treated, or does not respond to treatment, but enters a chronic state, people may live more of their lives with PTSD than the other NCDs mentioned.

### Main findings

Our study disclosed lower rates of trauma exposure compared to what has been reported in previous gender-specific epidemiologic studies, as summarized by Olff et al. [18]. When comparing incidence rates of traumatic exposure across studies and populations, one needs to consider the questionnaires applied and the events included in each. In addition, there are likely differences between populations regarding what constitutes a traumatic event [19]. It has been hypothesized [20] that the majority of events involved in survey instruments are traumas more likely to happen to men than to women. In our study, the proportion of men having experienced the trauma was greater in four out of nine trauma types. In addition, the M-CIDI includes an “other traumatic events” category, allowing respondents to include all experienced traumas.

Our results suggest an increased risk of subsequent PTSD for women having suffered from one or more psychiatric disorders prior to traumatic exposure. Had a measure of problematic drinking been included in “Any pre-existing psychiatric disorder,” the gender difference would probably have attenuated. Studies have shown that men to a greater extent than women attempt self-medication using alcohol against mental health problems [21]. A psychosocial explanation for the increased risk for PTSD in individuals with pre-existing psychiatric disorder would suggest that the conditions under which people with psychiatric illness may live either socioeconomically or in strained personal relationships, predispose them to increased chances for experiencing a traumatic event. Another explanation would indicate that being emotionally wounded puts you at risk of a traumatic event. Women are often considered more sensitive to interpersonal stress, with a higher sense of guilt [22]. In this scenario, the illness is related to personality factors that augment susceptibility to trauma. This warrants further study.

The fact that women more often than men fulfill diagnostic criteria for PTSD may be construed to mean that women are more vulnerable and less resilient. However, this may simply be a reflection of the fact that women and men are exposed to different types of potentially traumatic events. This study revealed that women more often were exposed to rape, sexual abuse as a child and verbal threats or violence from close relations. These are events associated with stigma and silence. Research on gender and coping has concluded that while men typically externalize, women typically internalize their reactions to stress [23]. Men tend to focus on changing the stressor, and women focus on changing how they react to the stressor. However, the gendered behavior pattern may be less important than the differences in the nature of traumas experienced. We hypothesize that unaddressed and untreated trauma more frequently will result in PTSD.

There is agreement in the literature that traumas of interpersonal violence are most likely to result in PTSD [24]. Our results support this. In accordance with previous research, e.g., [25], we found that men more often than women were exposed to PTEs. Accidental traumatic events, i.e., events not intentionally aimed at the individual (war, natural catastrophe, serious accident, witness trauma happen to others, or verbal threat/violence from non-close relation), resulted in PTSD nearly twice as frequent for men (*p* < 0.001). This may indicate that men are less risk-averse. Concurrently, there was no statistically significant difference to the frequency at which premediated traumas, i.e., events aimed at the individual suffering the trauma (physical threat with weapon, rape, sexual abuse, imprisonment/taken hostage/kidnapped, or verbal threat/violence from close relation), resulted in PTSD for women and men.

This study supported PTSD as a young adults’ disorder. While the incidence of trauma per 100,000 PYs sharply increased from age 40, the incidence of PTSD tapered off at the same age, most pronounced for men. Our findings contradict Dorrington et al. [25] who found reports of traumatic events to reduce in older age. Despite having a limited number of cases, our results do suggest that, particularly for men, resilience might be acquired through life experience and maturing.

Some differences were found in what traumatic events were endured at the rural and urban site, as well as different age of occurrence. These differences are difficult to explain. Living conditions do differ both in the social and professional contexts. The mentioned variations were too small to make assumptions about any significance, but do warrant further investigation.

The information uncovered here underlines the complexity of the PTSD diagnosis. The histories leading a trauma victim to become a PTSD patient come in multiple forms. Particularly among women, our results disclosed longer incubation time from trauma to PTSD. For both genders, the longevity of the PTSD exceeded the expected. According to ICD-10 diagnostic guidelines, PTSD is a diagnosis of transient character, typically not surpassing two years. It may well be the case that one, or more, less severe traumatic exposures, or life-events, later than the worst one, was the triggering event for the onset of PTSD. The unpleasant feelings resulting from the worst traumatization may be kept in control, as a subclinical state, until such a trigger event happened. This is in accordance with the human ability to relate new experiences with old ones, as described in Janet’s theory of dissociation [26]. Site-specific bodily memories of traumatic experiences may be reawakened decades later by rather innocent events, leading to diagnosable PTSD [27]. This is also a known phenomenon in the veteran’s literature [28]. Clinical experience shows that women who have been exposed to domestic violence, sexual abuse or rape may be exposed over a long period of time and are reluctant to and need a long time to disclose their situation and seek help. Such longstanding exposure and hesitation to help-seeking may give rise to a delayed development of PTSD [27]. We are aware of the controversy surrounding the PTSD diagnosis [25], both regarding potential diagnostic inflation [29] and the A criterion [30].

### Strengths and limitations

This study provides an estimate of the incidence and prevalence of PTSD in a western, general population. Most studies of post-disaster PTSD lack information about study participants’ pre-disaster mental health status. Hence, when such studies refer to ‘incidence,’ this should often rather be referred to as ‘prevalence’ at a post-disaster point of time. The use of the CIDI instrument in this study provided the opportunity of calculating incidence rates of both PTEs and PTSD per person year.

Stratifying on gender was a main objective of the present study. While much of the literature identifies differences between women and men on trauma exposure and PTSD incidence, most report results and forms their conclusions across genders, e.g., [5, 6, 25].

This study may include a small number of cases (exposed to trauma and PTSD). However, detailed information about each person may be a significant contribution to points of departure for further studies on coping, vulnerability, and prevention. Furthermore, it adds to the body of knowledge concerning future classification of PTSD in diagnostic systems. We are aware that work is underway toward including a diagnosis of complex post-traumatic stress disorder or DESNOS in ICD-11 [31].

Concerning the validity of the diagnoses, we can only refer to the excellent test–retest reliability of the CIDI internationally [14], and refer to the difficulties with doing validity studies because of the lack of a gold standard for the diagnoses.

This study population had a lower response rate among the young, especially men. As PTSD is a young person’s disorder, there was a need to adjust for this. This was done using inverse probability weighting, adjusting the sample to reflect the composition of the Norwegian population. This study population, as well as most epidemiological questionnaire/interview studies, has a slight selection bias toward being healthier than the general population [32]. Our results, therefore, likely underestimate the true incidence and prevalence. Any study design will favor the participation of those whose general health allows them the physical ability to participate, and who coped well enough with their trauma to participate. Some of those most severely affected may be deceased, or in a state of health not allowing them to complete the interview. As always, there is a risk of recall bias when asking respondents about the past. This will also act to bias our findings, as some respondents might fail to recall previous exposure to trauma and/or subsequent reactions comprising PTSD and the resulting estimates of incidence and prevalence will be biased in a negative direction. Contrary, recall bias may also overestimate findings, because trauma/non-trauma was decided retrospectively, and this judgement could likely be affected by the current psychopathological state.

The Norwegian context should be taken into consideration. Norway has not seen war on home-soil since 1940–1945, and has only sparingly been involved in UN peacekeeping operations and other war-like situations. During the lifetime of our study participants, up to end of follow-up, Norway saw no major terrorism, with exception of WWII (1940–1945). In addition, Norway has low rates of criminal violence. Natural catastrophes have largely been limited to avalanches and rock slides. There have been a number of shipwrecks and other industry related accidents throughout the period.

### Public health and clinical implications

Exposure to potentially traumatic events, and subsequent PTSD, is a public health challenge. Some traumatic events, e.g., natural catastrophes, occur randomly, and are non-preventable. However, other events, e.g., rape, are very much preventable. As shown in this study, the most harmful traumatic events are indeed the preventable ones. Having this knowledge, society should intensify efforts to prevent traumas and provide adequate follow-up to individuals exposed to trauma. Public health education needs to be further targeted at reducing stigma and silence regarding personal traumatic exposure. The potential for prevention and help in processing traumatic events to avoid PTSD may be vast for both women and men.

In encounters with a traumatized individual, crisis intervention should always include an assessment of the individual’s past history of mental health, not least because of the increased risk of developing PTSD after pre-existing mental health problems.
